# Effect of CFRP on Buckling Performance of Thin-Walled Cylindrical Steel Tanks with Dents and Corrosion

**DOI:** 10.1021/acsomega.5c10375

**Published:** 2026-01-23

**Authors:** Mahmut Kılıç, Ömer Karadağ, Fatma Merve Korucuk, Yasin Tizi, Nurullah Çınar, Dilek Biten, Mahyar Maali

**Affiliations:** † Department of Civil Engineering, Engineering Faculty, 37503 Ataturk University, 25240 Erzurum, Türkiye; ‡ Department of Civil Engineering, Faculty of Engineering and Architecture, 226840 Erzurum Technical University, 25050 Erzurum, Türkiye; § Maali Çelik Ar−Ge Danışmanlık, Müh, İnş, Taah, Tarım ve Hayvancılık Company, Atateknokent, 25030 Erzurum, Türkiye

## Abstract

Thin-walled steel cylindrical shells offer excellent strength-to-weight ratios but are highly sensitive to mechanical imperfections and corrosion-induced degradation. This study investigated the effects of dents and corrosion on the buckling behavior of 0.45 mm-thick thin-walled cylindrical steel shells, as well as the efficiency of carbon fiber-reinforced polymer (CFRP) strengthening under external pressure. Nine specimens were tested, including corroded samples exposed to 2.5% and 5% HCl solutions to simulate material deterioration. The results revealed that both corrosion and dents significantly reduced the buckling capacity and structural stability of the specimens. However, in comparison with earlier investigations on unstrengthened and partially strengthened specimens, the external CFRP layers substantially mitigated capacity losses, improving ultimate load, ductility, and postbuckling behavior. Complementary SEM analysis provided microstructural insight into the corrosion mechanism, illustrating the progressive surface degradation from localized pitting to widespread delamination at higher corrosion levels. Overall, CFRP confinement was confirmed as a highly effective retrofitting solution for restoring strength and stability in imperfect and corroded thin-walled steel shells.

## Graphical abstract

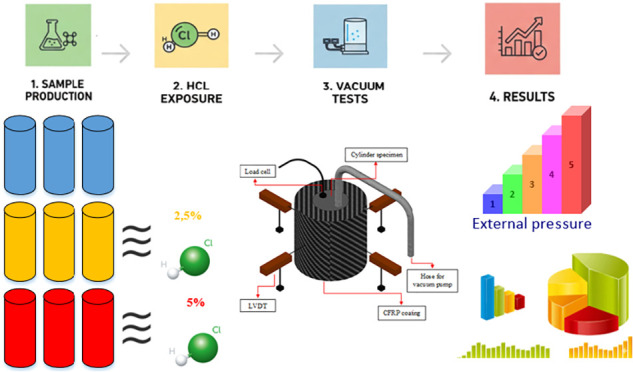

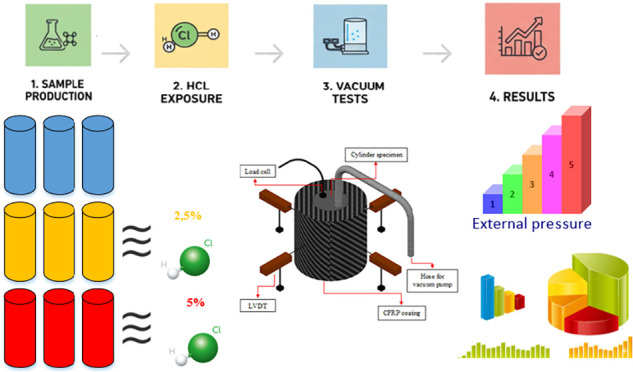

## Introduction

Thin-walled steel structures have become increasingly prevalent in modern engineering applications, particularly in the construction of tanks for liquid storage, due to their lightweight, high strength-to-weight ratios, and efficient material usage. These attributes make them ideal for applications where structural integrity and weight are critical considerations, though their buckling behavior under various loading conditions, such as pressures, remains a key challenge.

To enhance the stability of thin-walled steel tanks, various reinforcement techniques have been explored. One promising approach involves the use of Carbon Fiber Reinforced Polymer (CFRP) materials, which have been shown to significantly increase the load capacity of thin-walled steel elements. The application of CFRP reinforcement allows the steel shell to sustain loads beyond its buckling capacity.^1^ It has been demonstrated that the application of CFRP tapes can enhance the maximum load capacity of thin-walled steel beams by as much as 200%, depending on the placement method of the CFRP.^2^ Additionally, CFRP coatings can help mitigate the detrimental effects of corrosion on the buckling behavior of thin-walled cylindrical shells.^3^ Researchers have investigated the buckling and postbuckling behavior of dented cylindrical shells reinforced with CFRP strips under uniform external pressure.^4^ Moreover, the integration of composite materials, such as CFRP, into the design of thin-walled steel tanks has been shown to improve their performance under seismic loading conditions. Çelik et al.^5^ conducted studies that indicated the effectiveness of CFRP in strengthening cylindrical steel water tanks subjected to seismic forces, thereby improving their resilience against dynamic loads

The corrosion resistance of thin-walled steel is another critical factor influencing its application in tanks, especially in industrial environments. It has been reported that the surface characteristics of cold-formed thin-walled steel can degrade over time, necessitating protective measures to maintain structural integrity.^6^ Corrosion-induced wall thinning, combined with dents, significantly reduces the buckling and load-bearing capacity of structures.^7^

Dents are also a common type of geometric imperfection in thin-walled cylindrical shells, which can significantly impact their buckling behavior.^7–10^ The presence of dents can lead to stress concentrations and reduce the overall buckling strength of the structure.^11^ The buckling capacity of thin-walled cylindrical shells is strongly influenced by dent size, depth, location, and the “neighborhood effect” of closely spaced dents.^10–12^

Despite extensive investigations into the effects of dents, corrosion, and CFRP reinforcement individually, there remains a lack of studies systematically addressing their combined influence on thin-walled steel shells. Understanding these interactions is crucial not only for predicting buckling and failure behavior, but also for developing practical guidelines for design, maintenance, and repair of tanks and pipelines in industrial applications. The present study aims to bridge this gap by providing a comprehensive assessment of how CFRP reinforcement modifies the structural response under simultaneous geometric and material degradation.

Compared with prior studies, this work provides a clear advancement in evaluating the combined dent–corrosion behavior of thin-walled cylindrical steel shells. In study,^1^ CFRP reinforcement was examined only for perfect and uniformly corroded cylinders, without considering dent imperfections. Study,^7^ in contrast, investigated various dent depths together with corrosion levels, but did not include any CFRP strengthening, leaving the influence of CFRP on the dent–corrosion interaction unaddressed. The present study fills this gap by examining the same ranges of dent depths and corrosion levels used in the literature, but with all specimens strengthened using CFRP layers. This approach enables, for the first time, a direct assessment of how CFRP modifies buckling capacity and failure mechanisms under combined geometric and material degradation, thereby advancing the understanding beyond previous works.^1,7^

Fazlalipour et al.^13^ summarized advancements since 2005 on the buckling behavior of steel cylindrical shells under external pressure, showing that various reinforcement methods (stiffeners, corrugations, CFRP, thickness variation, confinement) and geometric imperfections (ovality, welding flaws, waviness, dents) strongly influence buckling resistance. They also showed that combined loading conditions—such as axial compression, bending, or internal pressure—significantly reduce the buckling capacity, emphasizing that understanding these interactions is essential for developing safer and more efficient cylindrical shell designs.

Nabati and Ghanbari-Ghazijahani et al.^14^ investigated circular steel tubes with cutouts strengthened by CFRP under axial compression, showing through experiments and numerical models that properly placed CFRP layers can shift stress concentrations away from critical regions and significantly recover the load-carrying capacity lost due to the cutout. They found that partial CFRP layouts, optimized in terms of fiber orientation and number of layers, were more effective and material-efficient than full wrapping, demonstrating that strategic CFRP placement can delay buckling, enhance capacity, and improve ductility while also offering practical advantages such as ease of installation, corrosion resistance, and improved fatigue performance.

Zhao et al.^15^ investigated the burst pressure and strain behavior of thin-walled pipes with dent and gouge defects through experimental and numerical methods. Their findings reveal that gouge size has the most significant impact on burst pressure, while dent depth, pipe diameter-to-wall-thickness ratio, and gouge depth and length also play critical roles. They suggest that future research should explore other defect types, such as punctures, corrosion, and cracks, as well as the effect of indenter type on pipeline burst pressure. Zhang et al.^16^ examined the collapse performance of composite-repaired cylinders with internal metal loss under external pressure through experimental and numerical methods. Results showed that composite reinforcement can fully restore and even enhance the external loading capacity of thinned cylinders, making them less sensitive to initial geometric imperfections than intact cylinders. Their findings highlight the effectiveness of composite repairs in improving the structural performance of damaged cylinders, with strong agreement between experimental and numerical analyses. Lin et al.^17^ studied the dynamic response and impact resistance of thin-walled FRP-concrete-steel tubular towers (TW-FCSTs) under horizontal impact loading through experimental and numerical analyses. Results indicated that increasing FRP and steel thickness enhances energy dissipation, reduces localized dent deformation, and improves structural stiffness, while higher void ratios lead to more localized denting and less overall deformation. Their finite element models accurately simulate the dynamic performance of TW-FCSTs, with parametric studies highlighting the effects of impact velocity, mass, and material properties on structural behavior. Chegeni et al.^18^ investigated the impact of corrosion depth and shape on the performance of thin-walled steel pipes subjected to combined internal pressure and bending load. The results showed that increased corrosion depth significantly reduces the bending capacity, while corrosion shape has less impact, with circumferential corrosion being the most detrimental. Their study concludes that while internal pressure does not affect the load-carrying capacity significantly until it exceeds a certain threshold, corrosion depth and shape play a critical role in the bending performance of corroded pipes. Yadav and Gerasimidis^19^ examined the buckling behavior and imperfection sensitivity of thin steel cylindrical shells under pure bending, focusing on the impact of geometric imperfections and strain-hardening models for slenderness ratios between 60 and 120. Their findings reveal that biased imperfections significantly reduce the load-carrying capacity and collapse curvature, while strain-hardening models, particularly the Ramberg-Osgood model, greatly influence bending capacity and curvature. Their results emphasize the critical role of imperfection type and material modeling in accurately predicting the structural behavior of cylindrical shells, offering valuable insights for the design of energy structures like wind turbine towers. Huang et al.^20^ developed a finite element-based multiphysical field coupling model to investigate the mechano-electrochemical interaction at dent-corrosion defects in X80 steel pipelines, accounting for the combined effects of curvature changes and wall thickness thinning. Their results show that dent depth, defect geometry, and diameter-to-thickness ratio significantly influence local strain and anodic current density, while operating pressure has a marginal effect; circumferential dents have a greater impact than axial dents. Their findings emphasize the need for integrated assessment methods for dent-corrosion defects, efficient inspection techniques, and further research into defect growth and failure mechanisms over time. Bastani et al.^21^ investigated the structural behavior of damaged steel I-beams rehabilitated with CFRP fabric under four-point bending loads. Their results indicated that the yield and ultimate load capacities, as well as the neutral axis depth, can be fully restored to the level of undamaged beams by using the appropriate number of CFRP layers while avoiding debonding failure, though full ductility restoration remains challenging. A validated finite element (FE) model predicted the required CFRP layers for different defect levels and successfully simulated the rupture behavior of CFRP fabrics. Hocine et al.^22^ reviewed methods for assessing and repairing corroded steel pipelines, focusing on the effectiveness of composite wrap repairs. They highlighted the advantages of synthetic composite materials, such as high strength, stiffness, and corrosion resistance, and explored optimization techniques to enhance repair performance and reduce costs, including the use of biocomposites for sustainable solutions. Their findings emphasize the need for improved design practices to balance safety, cost-efficiency, and environmental impact while proposing deterministic and reliable finite element models for evaluating failure pressures and optimizing repair configurations.

## 1 Experimental Study

### 1.1 Details of Specimens

The details of the specimens are presented in Table 1. Steel sheets with a thickness of 0.45 mm were utilized to fabricate the cylindrical steel shell specimens. To ensure the specimens demonstrated realistic behavior, the radius-to-thickness (*r*/*t*) ratios were chosen to range between 300 and 1000.^22^ Each cylindrical shell has a radius of 200 mm, a height of 400 mm, and an *r*/*t* ratio of 445, which was within the desired range for realistic testing conditions. The steel sheets were precision-cut to the required dimensions using CNC machining and then formed into cylindrical shapes. Steel plates, also with a radius of 200 mm, were used as covers, each having two perforations. One hole served to connect a vacuum pump, while the other was fitted with a load cell. A total of nine cylindrical shell specimens were prepared, consisting of three noncorroded specimens and six corroded specimens. Among the corroded samples, three were exposed to a 2.5% HCl solution, and the remaining three to a 5% HCl solution. The dent regions for each group of specimens, noncorroded, 2.5% corroded, and 5% corroded, were modified in a stepwise manner. The terms 1t, 2t, and 3t in the specimen names refer to the radius of the dent being examined in the specimen (*t*_*x*_). Here, the value of *t* represents the thickness of the cylinder (0.45 mm), and the dent sizes were created at values corresponding to the thickness, twice the thickness, and three times the thickness, respectively (Figure 1).

**Figure 1:**
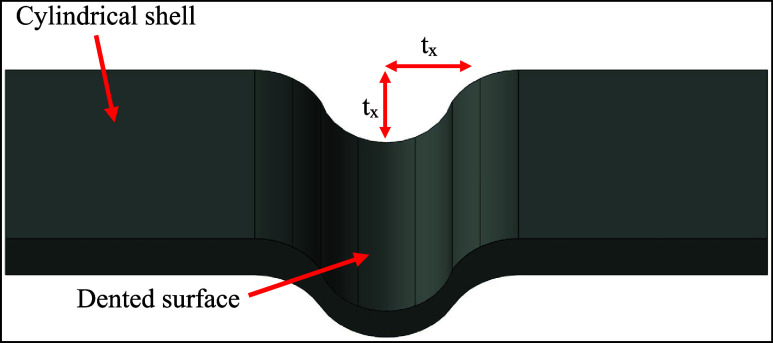
Dent size for dented specimens.

**Table 1:** Properties of the Experimental Specimens

			weight of the specimens (gram)			dimensions (mm)		
group	specimen	HCl Ratio (%)	before corrosion	after corrosion	weight loss ratio (%)	thickness	height	radius
noncorroded	CFRP-1t	-	-	-	-	0.45	400	200
	CFRP-2t		-	-	-			
	CFRP-3t		-	-	-			
corroded	2.5% CFRP-1t	2.5	1555	1447	6.9			
	2.5% CFRP-2t		1550	1444	6.8			
	2.5% CFRP-3t		1545	1443	6.6			
	5% CFRP-1t	5	1550	1404	9.4			
	5% CFRP-2t		1545	1401	9.3			
	5% CFRP-3t		1555	1402	9.8			

The tensile properties of the steel sheet material used for creating the cylindrical shells were evaluated by conducting three tensile tests on coupon samples, following the guidelines set by ASTM-E8m.^23^ The yield stress was calculated using the 0.2% offset method. The tensile strength was derived by dividing the highest force recorded during the test by the sample’s initial cross-sectional area. Young’s modulus was determined from the initial slope of the stress–strain curve, representing the tangent modulus within the elastic range. The average values of these mechanical properties are summarized in Table 2.

**Table 2:** Tensile Properties of Cylindrical Shell

Young’s modulus (GPa)	yielding stress (MPa)	ultimate stress (MPa)	Poisson’s ratio
**210.01**	198.82	342.44	0.29

In the experimental setup, a support for the laser measurement device was placed at a suitable distance from the specimen, selected from the grooved sections on the metal plate where the specimen was fixed using silicone (Figure 2). The purpose of this setup was to enable noncontact and accurate measurement of geometric imperfections on the surface of the cylindrical shell.

**Figure 2:**
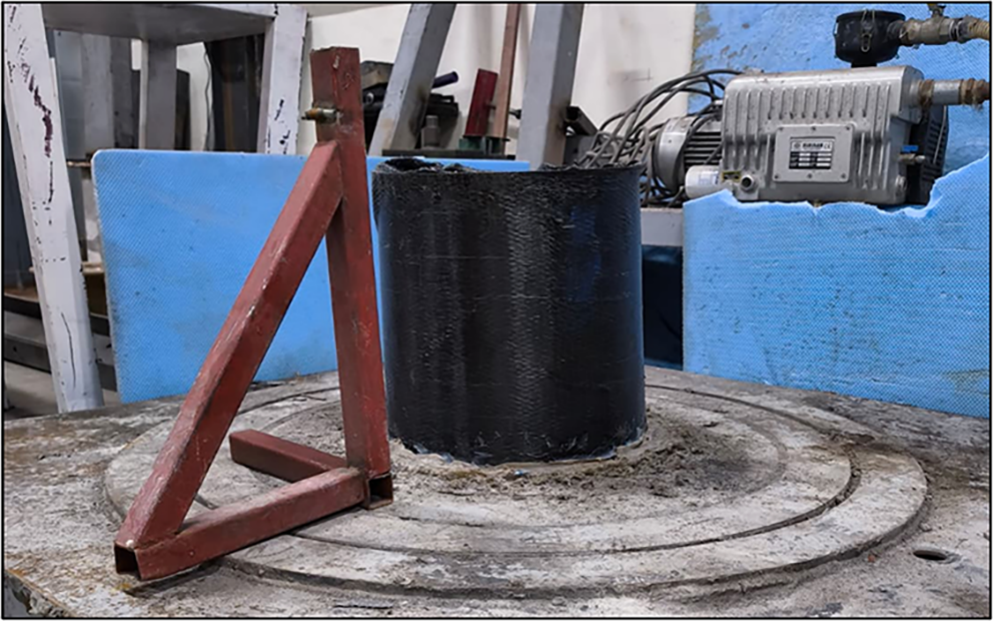
Test rig for measuring imperfections.

Figure 3 shows the radial geometric imperfections measured along the height of the cylindrical specimen. Measurements were taken at 25 circumferential mesh points at height levels of 50, 100, 150, 200, 250, 300, and 350 mm along the cylinder. The dashed red line represents the perfect circular shape, which serves as the reference. The other lines correspond to the actual measured profiles at the respective heights. The data are presented in polar coordinates: the angular positions (0 to 24) represent the circumferential mesh points, and the radial distances indicate the magnitude of imperfections in millimeters. Positive values denote outward deviations, while negative values indicate inward deviations. The measurement results were recorded, and the imperfection ratios of the specimen were calculated prior to loading. It was determined that the maximum deviations at the mesh points were approximately 5 mm. These irregularities were considered negligible, and thus, the experiments were conducted without further modification of the specimen. Although local fluctuations were observed, the overall geometric deviations remained within acceptable tolerances for the test program. Similar levels of initial imperfections have also been reported in previous studies, and were likewise deemed not to significantly influence the structural behavior.^1,7^

**Figure 3:**
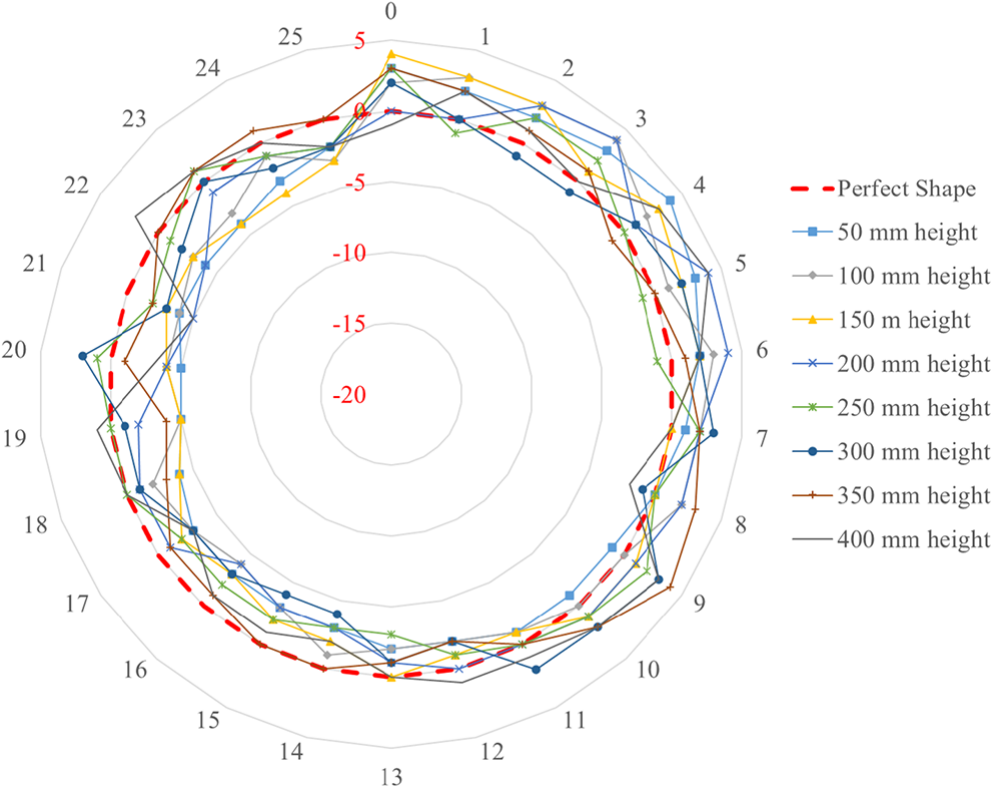
Observed geometric imperfections.

The setup for the acid exposure was the same as that employed in our previous studies.^1,7^ In brief, two hydrochloric acid solutions (2.5 % and 5 %) were prepared from reagent-grade HCl in accordance with TS-EN ISO 9001:2008.^24^ The stock acid had a declared purity of 30–32 % and a density between 1.15 and 1.16 g/cm^3^, with trace Fe and As contents of 0.0005 % and 0.0001 %, respectively. The concentrations of 2.5 % and 5 % HCl were chosen to represent mild and moderate corrosive environments commonly encountered in practice—such as descaling operations in chemical processing, exposure to acid rain in industrial regions, or storage of mildly acidic solutions in containment vessels; without inducing unmanageably rapid material degradation. Lower concentrations would yield negligible mass loss over short periods, while higher concentrations risk complete surface dissolution and unrealistic failure modes for thin-walled shells; 2.5 % and 5 % strike an optimal balance, producing measurable corrosion pits and uniform attack patterns suitable for comparative buckling studies.^1,3,7,25,26^ Specimens were fully immersed in each solution for 24 h to ensure that surface reactions reached near-steady-state conditions and produced sufficient weight loss for accurate quantification, while still preserving the shell geometry for structural testing. After 24 h immersion, specimens were rinsed in distilled water, gently dried, and weighed to determine mass loss.

Carbon fiber-reinforced polymer (CFRP) composites are widely used as an effective method for strengthening structural elements.^1^ The utilization CFRP in thin-walled structures offers enhanced strength-to-weight ratios, improved durability, and multifunctionality. CFRP exhibits exceptional tensile strength and stiffness, allowing for lighter designs without performance compromises, and provides superior fatigue and corrosion resistance for longer service life and lower maintenance.^27,28^ Applying CFRP materials to the outer surface of the shell is primarily preferred to eliminate the need for internal access and to minimize service interruptions.^29^ To ensure effective bonding and structural enhancement, the cleaned shell surfaces were coated with epoxy before applying a 230 g/m^2^ unidirectional carbon fiber fabric (Figure 4), selected for its high performance in structural reinforcement applications in Table 3.^30–32^

**Figure 4:**
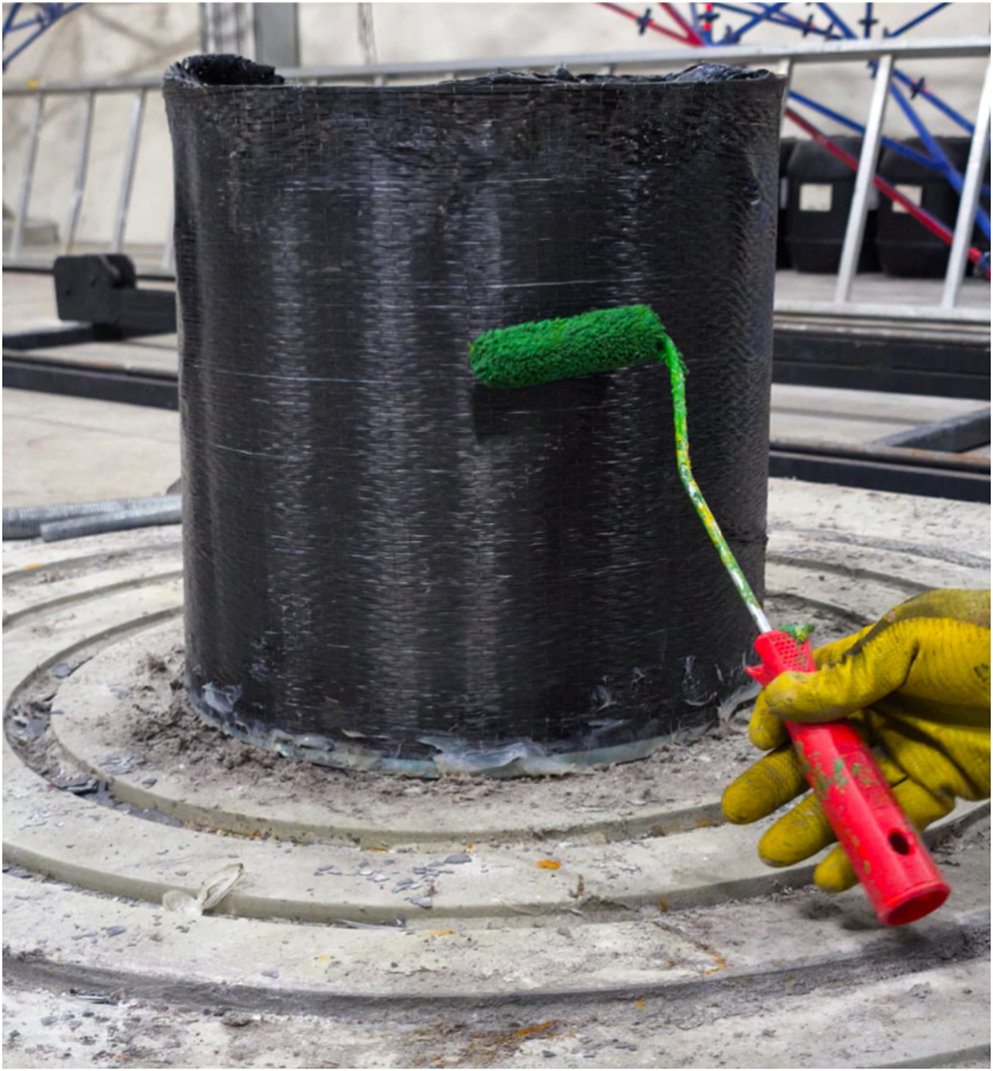
Epoxy coating and CFRP application on the specimen.

**Table 3:** Selected Mechanical and Physical Characteristics of the Carbon Fiber Fabric and Epoxy Resin^30–32^

property	carbon fiber fabric	epoxy resin
elastic modulus (MPa)	230,000	>2000
ultimate tensile strength (MPa)	4900	-
compressive strength (MPa)	-	>60
shear strength (MPa)	-	>6
flexural strength (MPa)	-	>50
nominal thickness (mm)	0.111	-
areal density (g/m^2^)	230	-
strain at failure (%)	2.10	-
material density (kg/L)	-	1.02
viscosity range (mPa·s)	-	1500–2500

### 1.2 Details of the Test Setup

The test setup employed in this study is illustrated in Figure 5. Each cylindrical specimen has a height and diameter of 400 mm, with a wall thickness of 0.45 mm. Uniform external pressure was applied to the specimen to simulate realistic loading conditions such as vacuum-induced collapse or external confinement. To introduce a controlled mechanical imperfection, a continuous longitudinal dent line was created along the height of the cylindrical shell. This dent was formed before CFRP strengthening and extended across the full height of the specimen. Subsequently, the entire curved surface of the dented shell was externally coated with a carbon fiber-reinforced polymer (CFRP) layer. This configuration allowed for the assessment of CFRP’s effectiveness in strengthening geometrically imperfect and potentially corroded structures. A cross-sectional view of the specimen (A–A) highlights the dent profile, which has a depth equal to the wall thickness (0.45 mm). The specimens were mounted onto a base metal plate with circular grooves and sealed with cold silicone to prevent air leakage during pressurization. This arrangement enabled the evaluation of the combined effects of dent imperfections and full-surface CFRP strengthening on the buckling resistance of thin-walled cylindrical shells.

**Figure 5:**
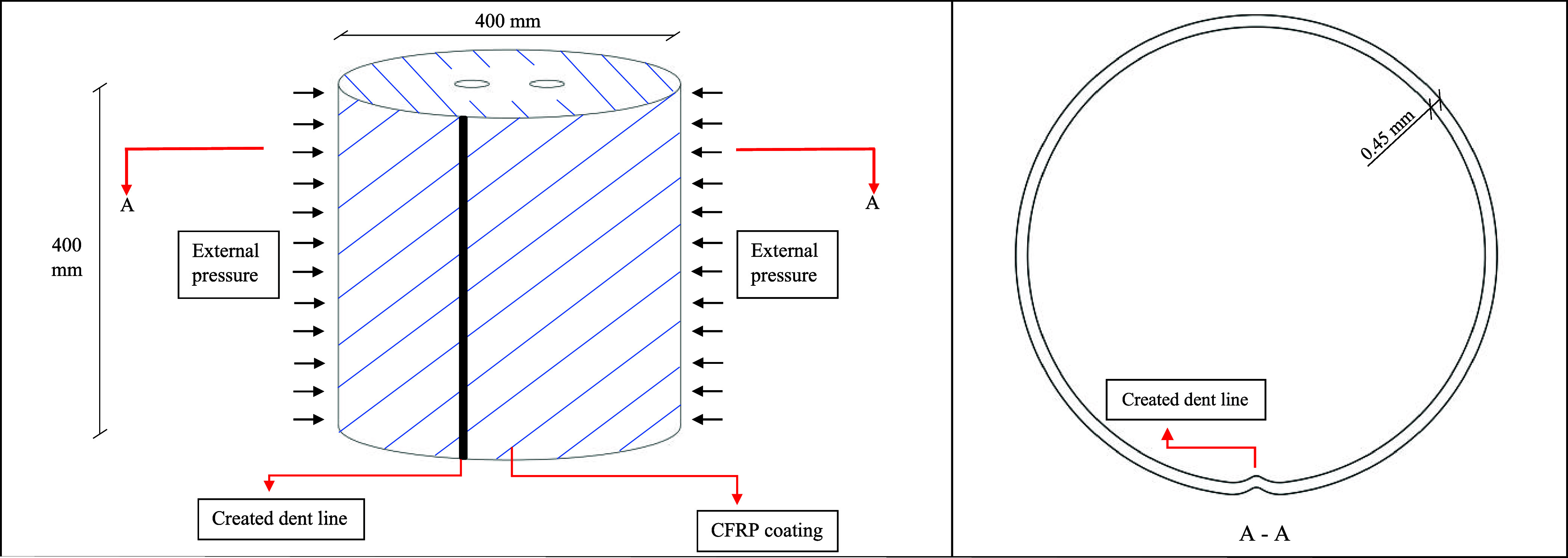
Schematic illustration of the test setup and cross-sectional view of the specimen.

The experimental test setup is shown in Figure 6. The cylindrical specimen was placed upright on a grooved metal plate and sealed using cold silicone to prevent air leakage during pressurization. A vacuum pump was connected to one of the openings on the top cover via a flexible hose to generate external pressure by reducing the internal pressure. The other opening was equipped with a load cell to monitor the internal pressure in real time. To measure the lateral deformations of the specimen under pressure, four Linear Variable Differential Transformers (LVDTs) were symmetrically positioned around the circumference at a height of 200 mm from the base. The measurements from the four LVDTs showed good consistency, indicating minimal variability and confirming the repeatability of the recorded deformations. As illustrated earlier in Figure 4, the entire curved surface of the cylindrical shell, including the dented region, was externally coated with carbon fiber-reinforced polymer (CFRP) fabric. This configuration allowed the evaluation of the CFRP’s strengthening effect under uniform external pressure.

**Figure 6:**
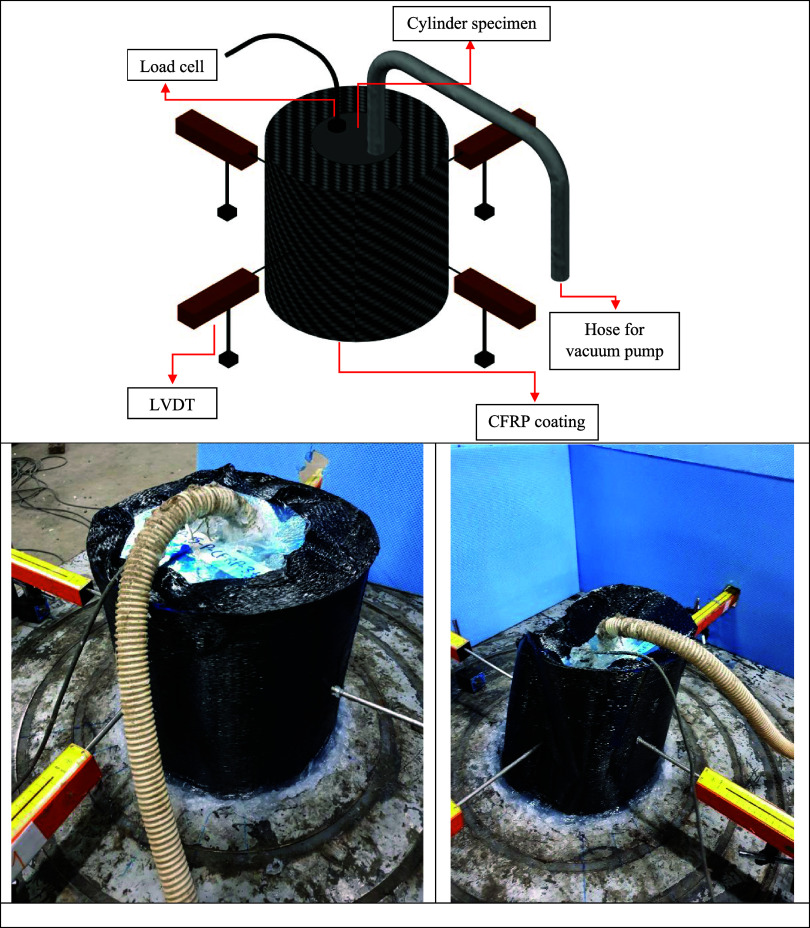
Test setup with CFRP-coated cylinder and vacuum system.

## 2 Results and Discussion

### 2.1 Buckling Response

The reported buckling loads represent the average of repeated measurements, and the small variations observed across the LVDTs confirm the repeatability and reliability of the experimental data.

Figure 7 presents the external pressure–displacement relationships obtained from the four LVDTs, providing detailed information on the deformation patterns until collapse, while Table 4 lists the characteristic buckling values, including the initial buckling, overall buckling, and collapse loads. In contrast to the unstrengthened shells studied previously,^7^ the application of CFRP significantly altered both the load-bearing capacity and the deformation modes. Total buckling waves reported in Table 4 correspond to the number of circumferential lobes observed in the deformed profiles of the cylindrical shells under external pressure, as illustrated in Figure 11. These lobes represent the local buckling patterns along the shell circumference, with the count reflecting the degree of wave propagation for each specimen.

**Table 4:** Load Parameters of Specimens

specimen	corrosion %	load of initial buckling (kPa)	load of overall buckling (kPa)	load of collapse buckling (kPa)	overall buckling load-to-initial buckling load	collapse load-to-overall buckling load	total buckling waves
CFRP-1t	-	2.95	6.9	9.90	2.34	3.36	5
CFRP-2t		2.85	7.13	17.54	2.50	6.15	4
CFRP-3t		2.53	17.75	26.35	7.02	10.42	1
2.5%-CFRP-1t	2.5%	2.69	7.29	19.61	2.71	7.29	2
2.5%-CFRP-2t		0.88	12.22	12.22	13.89	13.89	0
2.5%-CFRP-3t		2.63	18.69	18.69	7.11	7.11	2
5%-CFRP-1t	5%	1.62	20.39	22.42	12.59	13.84	4
5%-CFRP-2t		2.05	17.25	17.25	8.41	8.41	4
5%-CFRP-3t		2.29	12.15	24.73	5.31	10.80	4

**Figure 7:**
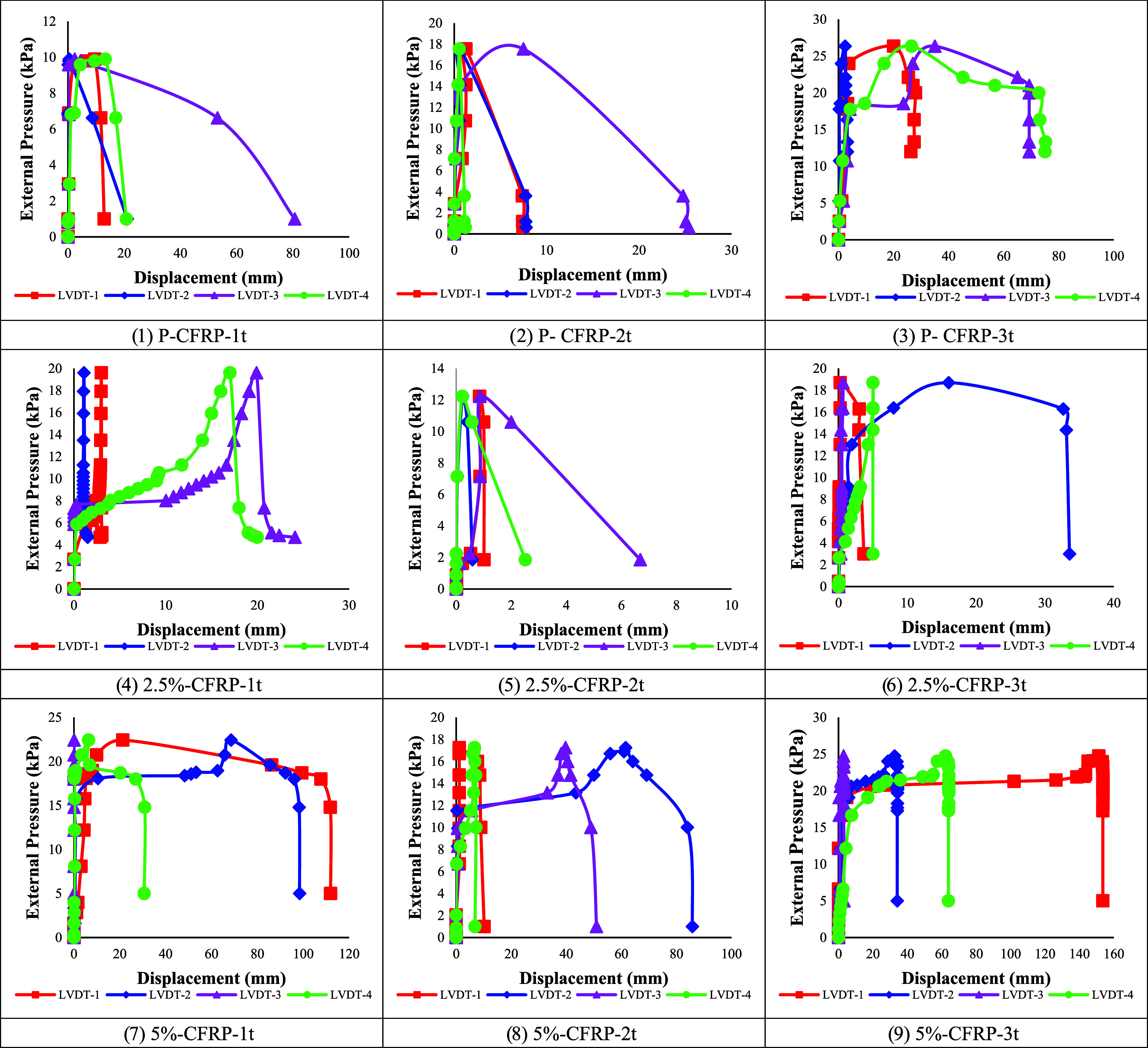
External Pressure-Displacement curves of CFRP-strengthened specimens.

The results show that the CFRP layers enhanced the overall stability of the cylindrical shells by delaying the onset of buckling and increasing the collapse loads. A similar strengthening trend was also observed for corroded specimens: although corrosion reduced the initial buckling resistance, the CFRP wrapping allowed these specimens to sustain relatively higher overall and collapse loads compared to their initial buckling levels. As summarized in Table 4, specimens with CFRP layers generally exhibited fewer total buckling waves, which is noticeably lower compared to the wave numbers observed in the previous study without CFRP strengthening.^7^ This reduction in wavenumber indicates that CFRP reinforcement constrained local instabilities and promoted more global buckling behavior.

Figure 8 compares these values across all specimens for a clearer evaluation. As the dent depth increased, initial buckling loads tended to decrease, while overall and collapse loads varied depending on the corrosion ratio. While dents and corrosion reduce the structural integrity of the shells, CFRP strengthening allows the specimens to sustain significantly higher overall and collapse loads despite these imperfections.

**Figure 8:**
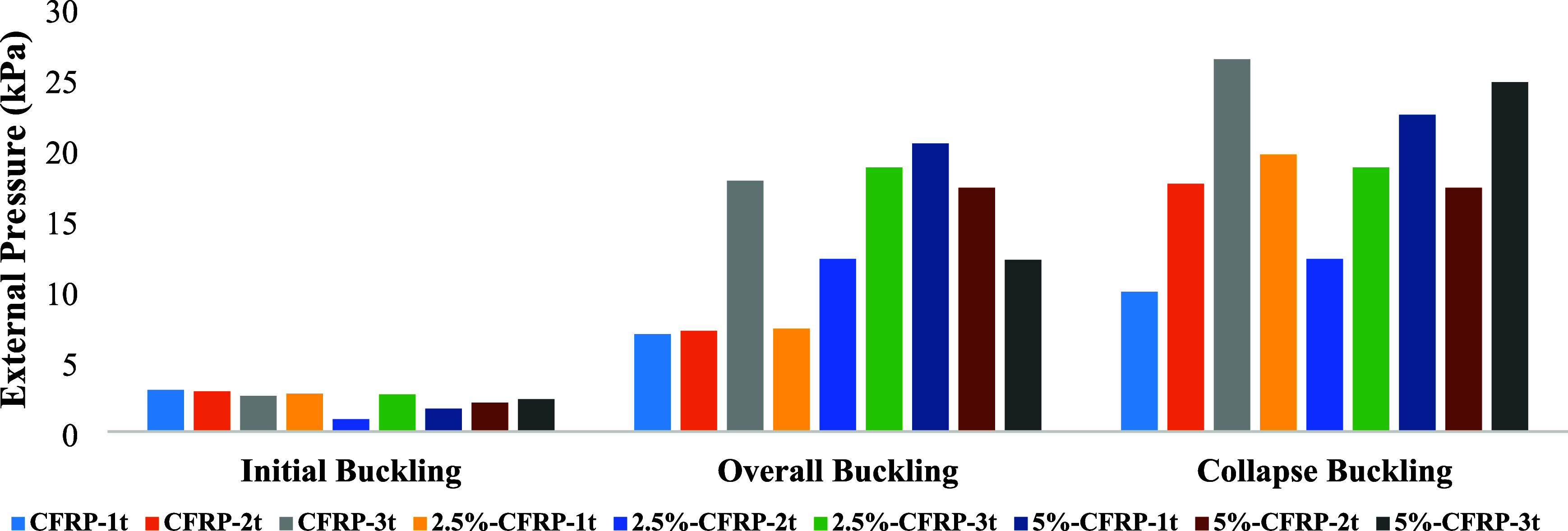
Comparison of buckling loads of specimens.

### 2.2 Effect of Dent and Corrosion on the Buckling Behavior of Specimens

For the noncorroded specimens, an increase in dent depth was generally associated with a decrease in initial buckling load, dropping from 2.95 kPa (CFRP-1t) to 2.53 kPa (CFRP-3t). However, despite this reduction in the onset of instability, specimens with larger dents demonstrated higher overall and collapse capacities. In particular, CFRP-3t achieved the maximum collapse load of 26.35 kPa, more than 2.5 times that of CFRP-1t. This indicates that while deeper dents promote earlier local buckling, the CFRP layers restrain further propagation and enable the specimens to carry higher loads before collapse.

Corrosion exhibited a different influence. At 2.5% corrosion, initial buckling loads were noticeably reduced, most significantly for 2.5%-CFRP-2t (0.88 kPa), which recorded the lowest value among all specimens. Nevertheless, some corroded specimens displayed competitive or even higher collapse loads compared to noncorroded cases. For instance, 2.5%-CFRP-1t reached a collapse load of 19.61 kPa, nearly double that of its noncorroded counterpart (9.90 kPa). This suggests that localized corrosion altered the initiation of buckling but did not necessarily compromise the ultimate load resistance once CFRP confinement became dominant. At the higher corrosion level (5%), the trend was more pronounced. While the initial buckling values were further lowered (e.g., 1.62 kPa for 5%-CFRP-1t), some specimens still reached remarkably high collapse loads. Notably, 5%-CFRP-1t and 5%-CFRP-3t exhibited collapse loads of 22.42 and 24.73 kPa, respectively, surpassing several noncorroded specimens.

The external CFRP layer provided substantial confinement, which compensated for both dent-induced imperfections and material loss from corrosion, thereby delaying global collapse despite early local instabilities.

### 2.3 Comparison with Theoretical Formulations

To validate the experimental findings, the collapse buckling loads were compared with the values obtained from existing theoretical formulations.

1 $$P_{c}=\frac{2.6E{\left(\frac{t}{2r}\right)}^{2.5}}{\frac{h}{2r}-0.45{\left(\frac{t}{2r}\right)}^{0.5}}$$

2 $$P_{c}=\frac{0.92E{\left(\frac{t}{r}\right)}^{2.5}}{\frac{h}{r}}$$

*P*_c_: Buckling load *E*: Young’s modulus *t*: Wall thickness *r*: Radius *h*: Height

The classical expression proposed by Ross^33^ (eq 1) yielded a collapse load of 23.53 kPa, while Jawad’s formulation^34^ (eq 2) provided a similar value of 23.20 kPa.

When compared to the experimental results (Table 5 and Figure 9), it is evident that the theoretical predictions are consistently higher than most of the measured collapse loads. This discrepancy arises primarily from the fact that the analytical equations assume ideal cylindrical shells without considering the influence of geometric imperfections or material degradation. In the present study, all specimens included a continuous longitudinal dent as well as varying levels of corrosion, which are known to significantly reduce the effective buckling capacity. The dent promoted early local instability, while corrosion weakened the shell wall by reducing thickness and stiffness, thus lowering the load-carrying capacity.

**Figure 9:**
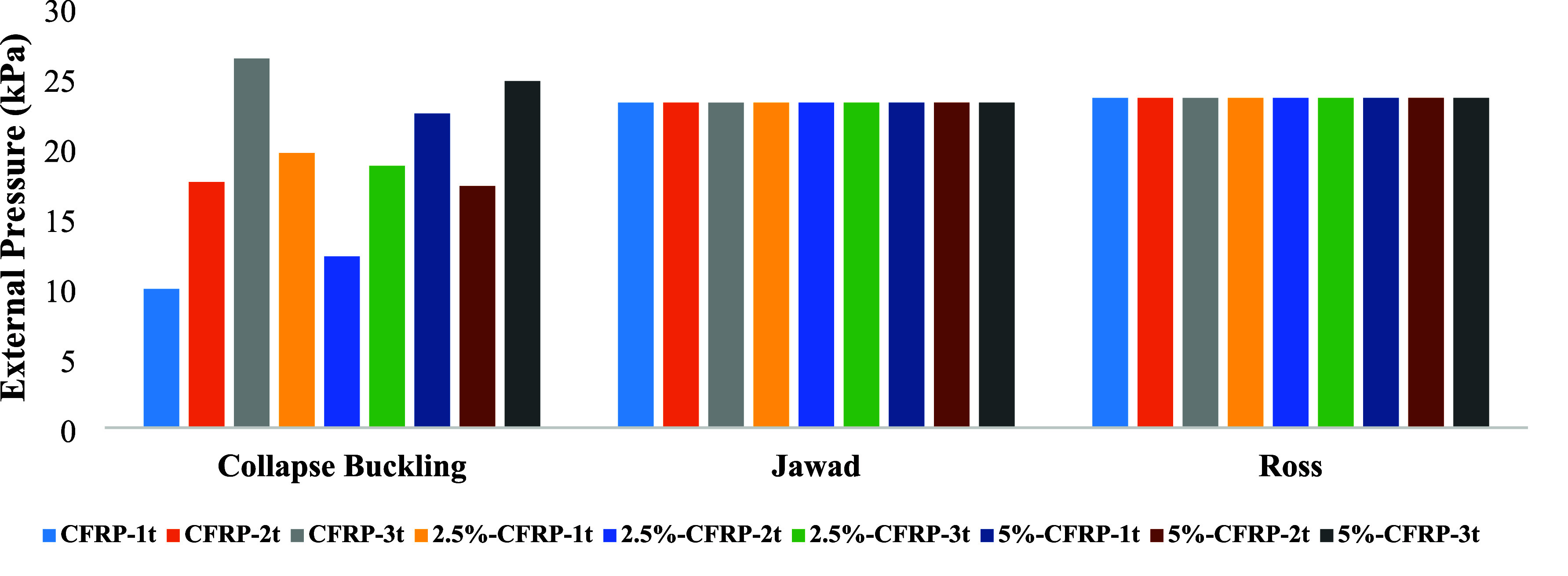
Comparison of experimental and theoretical collapse loads.

**Table 5:** Theoretical vs. Experimental Collapse Loads

specimen	collapse buckling (kPa)	Ross’ relationship (kPa)^32^	Jawad’s relationship (kPa)^33^
P-cfrp-1t	9.90	23.53	23.20
P-cfrp-2t	17.54		
P-cfrp-3t	26.35		
2.5%-cfrp-1t	19.61		
2.5%-cfrp-2t	12.22		
2.5%-cfrp-3t	18.69		
5%-cfrp-1t	22.42		
5%-cfrp-2t	17.25		
5%-cfrp-3t	24.73		

### 2.4 Failure Modes in Cylindrical Specimens

Lateral deformation patterns of the CFRP-strengthened cylindrical specimens, measured by four LVDTs (Figure 6), reveal the combined effects of dent depth and corrosion on buckling behavior. Specimens with deeper dents (CFRP-3t) showed larger local displacements initially, indicating early local buckling, yet CFRP layers effectively restrained these deformations and delayed global collapse. As summarized in Table 4, the number of buckling waves decreased with stronger CFRP confinement: CFRP-1t exhibited 5 waves, while CFRP-3t formed only 1 dominant wave, compared to higher wave counts in unstrengthened shells. Corrosion further promoted early localized bulging at dented regions (2.5% and 5%), reducing initial buckling loads, but CFRP maintained overall integrity and limited secondary waves. Consequently, even heavily corroded specimens (e.g., 5%-CFRP-1t) sustained high collapse loads with fewer waves. These results confirm that CFRP strengthening significantly modifies deformation modes, suppresses local instabilities, and enhances the load-carrying capacity of thin-walled cylindrical shells under external pressure. Figure 10. shows the failure modes of the CFRP-strengthened cylindrical specimens, while Figure 11. illustrates the overall deformation patterns of the cylindrical shells under external pressure.

**Figure 10:**
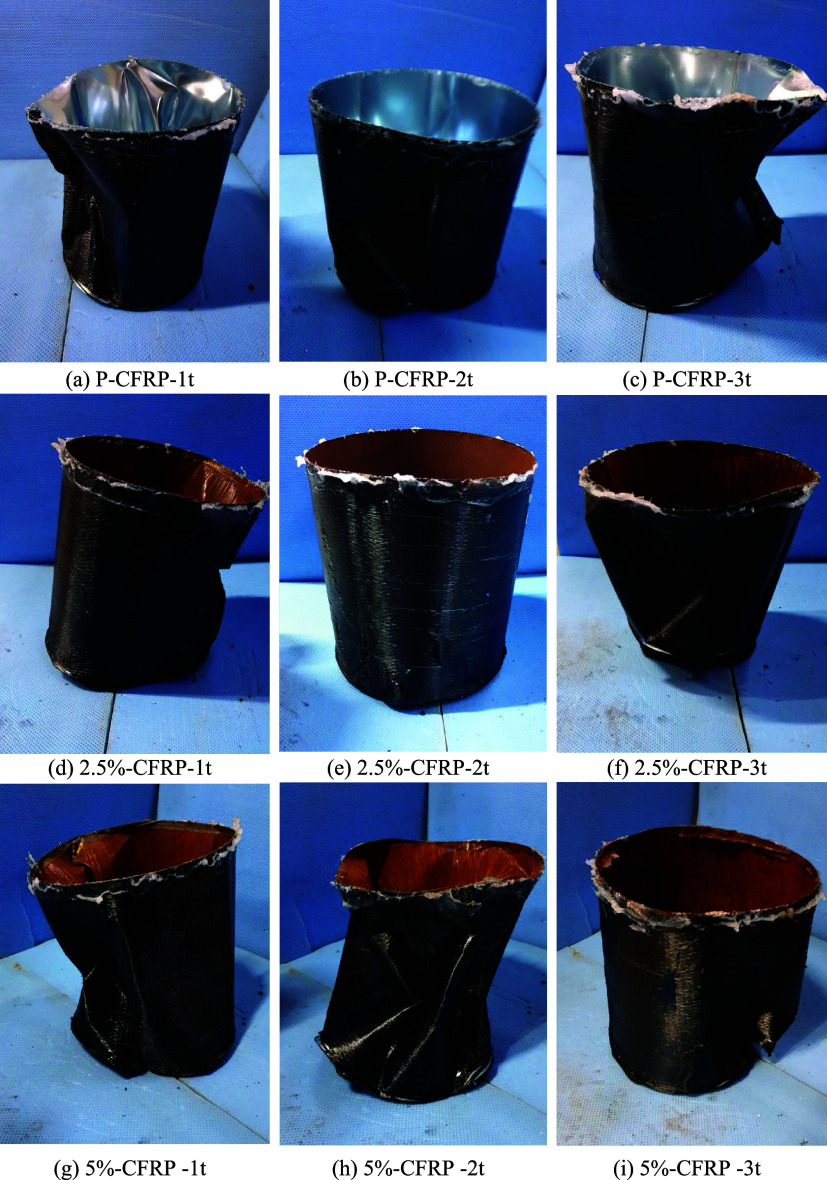
Failure modes of the specimens.

**Figure 11:**
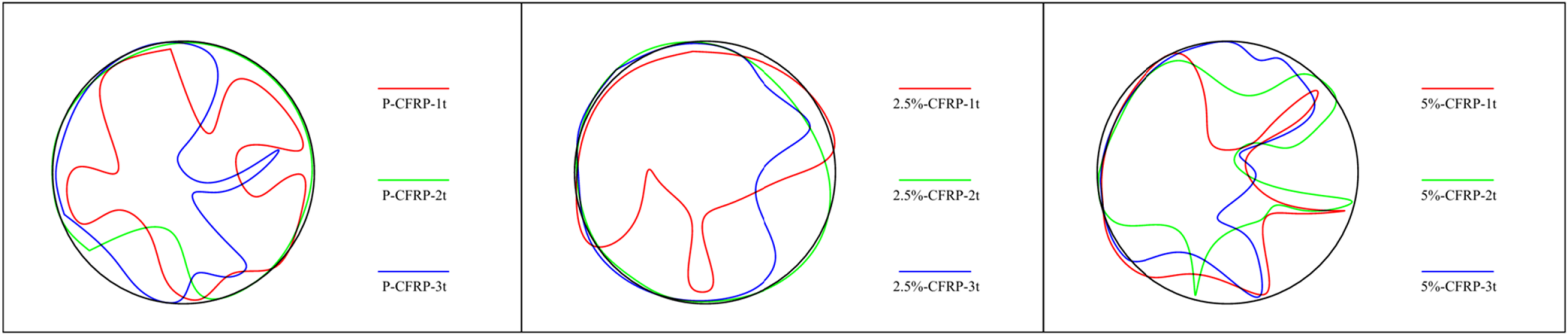
Radial deformations of cylindrical shells.

### 2.5 Comparison with Previous Studies

To contextualize the findings of the current study, the collapse buckling loads of CFRP-strengthened cylindrical specimens were compared with those reported in Previous Study No 1 ^7^ and Previous Study No 2 ^1^ in Table 6. In Previous Study No 1, strengthened specimens exhibited relatively low collapse loads, ranging from 3.65 to 11.14 kPa depending on dent depth and corrosion level. Previous Study No 2 included both strengthened and CFRP-strengthened specimens, showing that CFRP application increased collapse loads significantly compared to the strengthened counterparts, with values ranging from 5.33 to 23.11 kPa. In the present study, the CFRP-strengthened specimens consistently displayed higher collapse loads across all dent depths and corrosion levels, highlighting the effectiveness of the strengthening technique. For instance, P-CFRP-3t reached 26.35 kPa, substantially exceeding the corresponding values in Previous Study No 1 (P-3t: 9.48 kPa) and slightly higher than those in Previous Study No 2 (CFRP: 23.11 kPa). Similarly, corroded specimens, such as 2.5%-CFRP-1t and 5%-CFRP-3t, achieved collapse loads of 19.61 and 24.73 kPa, respectively, which are markedly higher than the loads reported for similar conditions in both previous studies. These comparisons indicate that the present experimental setup, combined with the full-surface CFRP reinforcement, effectively enhances the structural performance of thin-walled cylindrical shells, compensating for both dent-induced imperfections and material degradation due to corrosion. The results also suggest that differences in specimen preparation, dent geometry, and CFRP application methods likely contribute to the observed variations in collapse load values between studies.

**Table 6:** Comparison of Buckling Capacity for Dented and Dent-Free Specimens

current study		previous study no 1.^7^		previous study no 2.^1^	
specimen	collapse buckling (kPa)	specimen	collapse buckling (kPa)	specimen	collapse buckling (kPa)
P-cfrp-1t	9.90	P-1t	8.62	P	12.38
P-cfrp-2t	17.54	P-2t	9.14	CFRP	23.11
P-cfrp-3t	26.35	P-3t	9.48	2.5%-P	10.66
2.5%-cfrp-1t	19.61	2.5%-1t	11.14	2.5%-CFRP	17.43
2.5%-cfrp-2t	12.22	2.5%-2t	10.13	5%-P	5.33
2.5%-cfrp-3t	18.69	2.5%-3t	9.12	5%-CFRP	18.49
5%-cfrp-1t	22.42	5%-1t	4.67		
5%-cfrp-2t	17.25	5%-2t	4.05		
5%-cfrp-3t	24.73	5%-3t	3.65		

## 3 SEM Images of the Specimen Surfaces

SEM images of specimens under noncorroded, 2.5% corroded, and 5% corroded surfaces respectively are shown in Figure 12. The progression of corrosion on the surfaces of thin-walled cylindrical steel shells was examined through SEM to investigate the microstructural alterations induced by corrosion and their implications for mechanical performance. The SEM micrographs were obtained from three representative surface conditions: noncorroded, 2.5% corroded, and 5% corroded specimens.

**Figure 12:**
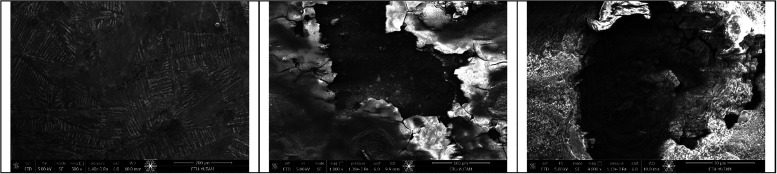
SEM images of specimens under noncorroded, 2.5% corroded, and 5% corroded surfaces, respectively.

The noncorroded specimen exhibits a compact, smooth, and homogeneous surface morphology, representing the intact microstructure of the steel. The uniform and featureless texture indicates a stable oxide film and the absence of any corrosion-induced deterioration, serving as a reliable reference for evaluating subsequent material degradation.

In contrast, the surface of the 2.5% corroded specimen displays the onset of corrosive attack, characterized by localized pitting, shallow surface irregularities, and the formation of microcracks. The severity and frequency of pits increased with higher corrosion levels, with 5% corroded specimens exhibiting visibly larger and more frequent pits compared to the 2.5% specimens. These features reflect the initiation phase of corrosion, where the passive protective layer begins to deteriorate, leading to localized metal dissolution. The presence of discrete corrosion sites suggests the establishment of anodic and cathodic regions that facilitate further propagation of corrosion under continued exposure.

The 5% corroded specimen shows the most pronounced degradation, marked by severe surface roughness, and interconnected microcracks. The microstructure reveals that the transition from localized pitting to generalized surface corrosion. The continuity of the metallic surface is disrupted, which directly compromises the load-bearing capacity and accelerates structural weakening under external pressure.

## 4 Conclusion

This study comprehensively investigated the collapse buckling behavior of thin-walled cylindrical steel shells with geometric imperfections and varying corrosion levels, strengthened externally with carbon fiber-reinforced polymer (CFRP) layers. Experimental results demonstrated that the application of CFRP confinement significantly enhances the structural performance of the shells, increasing both overall stability and ultimate load-carrying capacity under uniform external pressure.

The presence of dents and corrosion had a pronounced effect on initial buckling behavior, promoting earlier local instabilities and reducing early stage stability. Nevertheless, the CFRP layers effectively restrained these local deformations and limited the propagation of instabilities. As a result, specimens were able to sustain higher overall and collapse loads despite the presence of geometric imperfections and material degradation.

Load–displacement measurements indicated that CFRP strengthening altered the deformation modes of the cylindrical shells. The postbuckling responses were smoother, reflecting more uniform stress redistribution, while the number of buckling waves was reduced compared to unstrengthened specimens. Stronger CFRP confinement contributed to higher ductility and improved energy absorption capacity, demonstrating the role of CFRP not only in increasing collapse loads but also in enhancing structural resilience against both local and global buckling mechanisms.

Additionally, the SEM analyses provided microstructural evidence supporting the macroscopic findings. The progression from smooth and intact surfaces in noncorroded specimens to severely pitted and cracked morphologies in 5% corroded specimens revealed the direct influence of corrosion on material degradation. The SEM micrographs confirmed that corrosion leads to the breakdown of the steel surface, promoting localized pitting and grain boundary attack, which ultimately compromise the structural integrity of the shell. These observations correlate with the experimental results, where increased corrosion levels were associated with reduced stability and earlier onset of buckling. The presence of CFRP confinement is thus critical not only for enhancing load-bearing performance but also for mitigating the detrimental effects of corrosion on the steel surface.

Comparisons with theoretical predictions highlighted that idealized models overestimate collapse loads because they neglect geometric imperfections, corrosion effects, and boundary condition deviations. Furthermore, comparisons with previous studies confirmed that CFRP strengthening substantially improves performance relative to unstrengthened or partially strengthened shells. The combination of full-surface CFRP coverage and structural confinement proved critical in compensating for reductions in capacity caused by dents and material loss.

In summary, externally bonded CFRP provides an effective retrofitting strategy for thin-walled cylindrical shells affected by geometric imperfections and corrosion. The study emphasizes the importance of considering imperfection sensitivity, environmental degradation, and reinforcement measures when designing or retrofitting thin-walled structures. The results offer valuable guidance for improving the performance, durability, and safety of cylindrical shells in practical engineering applications.

These findings have practical engineering implications: they highlight the effectiveness of CFRP strengthening in compensating for dent and corrosion-induced weakening, providing guidance for retrofitting thin-walled steel shells. However, the study is limited to a single CFRP thickness and configuration, suggesting that further investigation is needed to optimize layer number, orientation, and material properties for different structural conditions.
